# Supplementary material for: High performance temperature difference triboelectric nanogenerator
Source: Nat Commun. 2021 Aug 6;12:4782. doi: 10.1038/s41467-021-25043-2 (PMC8346487; doi:10.1038/s41467-021-25043-2)
Supplement: Supplementary file 1 — Supplementary Information [file 41467_2021_25043_MOESM1_ESM.pdf]

Supplementary Information

High performance temperature difference triboelectric nanogenerator

Bolang Cheng[1], Qi Xu[1], Yaqin Ding[1], Suo Bai[1], Xiaofeng Jia[1], Yangdianchen Yu[1,2], Juan Wen[1]* & Yong Qin[1]*

[1] Institute of Nanoscience and Nanotechnology, School of Materials and Energy, Lanzhou University, Lanzhou 730000, China.

[2] Department of Material Science and Engineering, College of Engineering, Boston University, Boston, MA 02215, USA.

These authors contributed equally: Bolang Cheng, Qi Xu, Yaqin Ding

* email: wenj@lzu.edu.cn; qinyong@lzu.edu.cn

Supplementary Note 1

As shown in **Fig. Note 1**, an ideal temperature difference triboelectric nanogenerator with controllable friction layer temperature (TDNG) model can be built by ignoring the influence of borders and assuming that all transferred charge are concentrated on the surface of the friction layer (Kapton)[1]. The total charges in Al are $\sigma_{TDNG}A$-Q ($\sigma_{TDNG}A$ is the triboelectric charges (σ_{TDNG} is the surface charge density of TDNG, A is the surface area of TDNG), and -Q is the transferred charges between two electrodes).

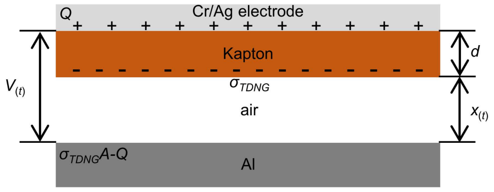

Fig. Note 1 The theoretical model of TDNG.

The $V_{(t)}$-Q-$x_{(t)}$ relationship of TDNG can be established as follows

$$V_{(t)} = U_{Kapton} + U_{air} = -\frac{Q}{A\varepsilon_0}\left(\frac{d}{\varepsilon_r} + x_{(t)}\right) + \frac{\sigma_{TDNG}}{\varepsilon_0}x_{(t)} \quad \text{(S1)}$$

where d is the thickness of the friction layer, ε_r is the permittivity of the Kapton, ε_0 is the permittivity of the vacuum, t is the time, and $x_{(t)}$ is the distance between two friction layers. Connected to an arbitrary resistance R, the output properties can be estimated by combining Ohm's law

$$V_{(t)} = I_{(t)}R = R\frac{dQ}{dt} \quad \text{(S2)}$$

Merging equation (S1) and equation (S2), we can have

$$R\frac{dQ}{dt} + \frac{Q}{A\varepsilon_0}\left(\frac{d}{\varepsilon_r} + x_{(t)}\right) = \frac{\sigma_{TDNG}}{\varepsilon_0}x_{(t)} \quad \text{(S3)}$$

with the boundary condition of when t = 0, Q = 0, this equation can be solved analytically as

$$Q_{(t)} = \sigma_{TDNG}A\left[1 - \left(1 + \frac{d}{RA\varepsilon_0\varepsilon_r}\int_0^t e^{\frac{1}{RA\varepsilon_0}\left(\frac{d}{\varepsilon_r}t + \int_0^t x_{(t)}dt\right)}dt\right)e^{-\frac{1}{RA\varepsilon_0}\left(\frac{d}{\varepsilon_r}t + \int_0^t x_{(t)}dt\right)}\right] \quad \text{(S4)}$$

Therefore, the current output can be derived by dQ/dt as follows

$$I_{(t)} = \frac{\sigma_{TDNG}d}{R\varepsilon_0\varepsilon_r}\left[\left(d + x_{(t)}\varepsilon_r\right)\left(\frac{1}{d} + \frac{\int_0^t e^{\frac{1}{RA\varepsilon_0}\left(\frac{d}{\varepsilon_r}t + \int_0^t x_{(t)}dt\right)}dt}{RA\varepsilon_0\varepsilon_r}\right)e^{-\frac{1}{RA\varepsilon_0}\left(\frac{d}{\varepsilon_r}t + \int_0^t x_{(t)}dt\right)} - 1\right] \quad \text{(S5)}$$

According to Ohm's law, the voltage with external load can be derived as follows

$$V_{(t)} = \frac{\sigma_{TDNG} d}{\varepsilon_0 \varepsilon_r}\left[\left(d + x_{(t)}\varepsilon_r\right)\left(\frac{1}{d} + \frac{\int_0^t e^{\frac{1}{RA\varepsilon_0}\left(\frac{d}{\varepsilon_r}t + \int_0^t x_{(t)}dt\right)}dt}{RA\varepsilon_0\varepsilon_r}\right)e^{-\frac{1}{RA\varepsilon_0}\left(\frac{d}{\varepsilon_r}t + \int_0^t x_{(t)}dt\right)} - 1\right] \quad \text{(S6)}$$

According to the relationship between the transferred charge density and temperature difference, the equation of voltage and current with external load can be described as follows

$$V_{(t)} = \frac{\left(-C_1\frac{\Delta T + b}{1-a} + C_2\right)de^{-SAt_0}}{\varepsilon_0 \varepsilon_r}\left[\left(d + x_{(t)}\varepsilon_r\right)\left(\frac{1}{d} + \frac{\int_0^t e^{\frac{1}{RA\varepsilon_0}\left(\frac{d}{\varepsilon_r}t + \int_0^t x_{(t)}dt\right)}dt}{RA\varepsilon_0\varepsilon_r}\right)e^{-\frac{1}{RA\varepsilon_0}\left(\frac{d}{\varepsilon_r}t + \int_0^t x_{(t)}dt\right)} - 1\right] \quad \text{(S7)}$$

$$I_{(t)} = \frac{\left(-C_1\frac{\Delta T + b}{1-a} + C_2\right)de^{-SAt_0}}{R\varepsilon_0 \varepsilon_r}\left[\left(d + x_{(t)}\varepsilon_r\right)\left(\frac{1}{d} + \frac{\int_0^t e^{\frac{1}{RA\varepsilon_0}\left(\frac{d}{\varepsilon_r}t + \int_0^t x_{(t)}dt\right)}dt}{RA\varepsilon_0\varepsilon_r}\right)e^{-\frac{1}{RA\varepsilon_0}\left(\frac{d}{\varepsilon_r}t + \int_0^t x_{(t)}dt\right)} - 1\right] \quad \text{(S8)}$$

Supplementary Figures

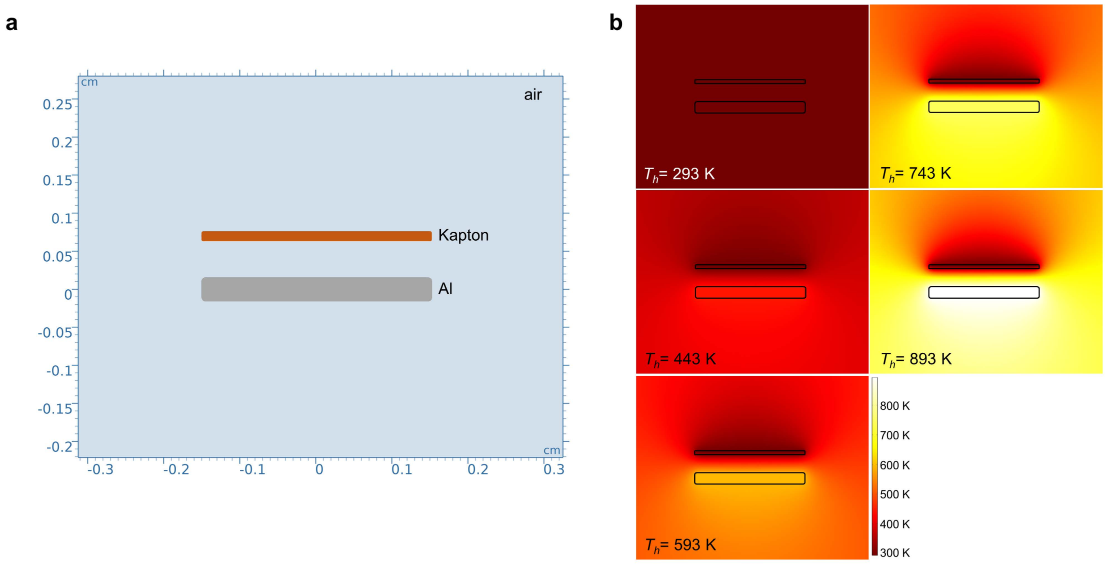

Supplementary Fig. 1 The model and temperature changes of TDNG. a The two dimensional (2D) geometric model of TDNG in COMSOL simulation. The simulation in Fig.1b and Fig.1c is independent except using the same 2D geometric model. Because of the convergence difficulties and deviation of the COMSOL program when simulating slab materials with a length/thickness ratio larger than 100 [2], the thickness of the Kapton friction layer is set as 10 μm and the Al friction layer's thickness is set as 30 μm, respectively; the width of Kapton friction layer and Al friction layer are set as 300 μm. The temperature change of the cooler friction layer (the inset in the right upper corner Fig. 1b) is obtained by steady simulation, which uses the module of heat transfer in solids and fluids in COMSOL. The thermal conductivity of Kapton and Al are set as 0.12 W m^{-1} K^{-1} and 238 W m^{-1} K^{-1}, respectively. Additionally, the Kapton and Al are both wrapped in an air domain, whose thermal conductivity is 0.023 W m^{-1} K^{-1}. Besides, the heat capacity of Kapton, Al, and air are set as 1090 J kg^{-1} K^{-1}, 900 J kg^{-1} K^{-1}, and 1.005 J kg^{-1} K^{-1}, respectively. The density of Kapton, Al, and air are set as 1430 kg m^{-3}, 2700 kg m^{-3}, and 1.293 kg m^{-3}, respectively. **b** The temperature distribution of TDNG with different temperature differences between the friction layer and the cooler friction layer. Through parametric sweeping, when the temperature of the hotter friction layer changes from 293 K to 893 K, the temperature distribution and the temperature change of the cooler friction layer can be obtained (the inset in the right up corner in Fig. 1b), and the temperature difference between the hotter friction layer and cooler friction layer can be obtained.

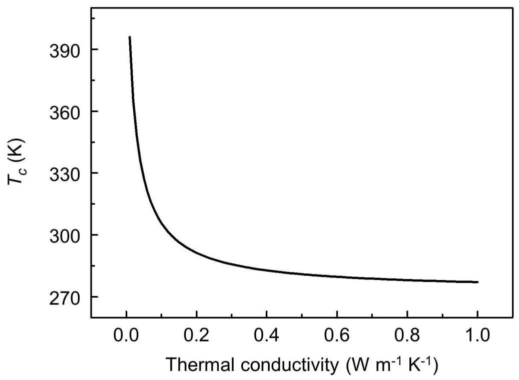

Supplementary Fig. 2 The relationship the cooler friction layer's temperature T_c and its thermal conductivity when the temperatures of heating system and cooling system keep at 593.15 K and 283.15 K, respectively. In TDNG, the cooler friction layer is closely attached to a cooling system. T_c is strongly influenced by the cooling system. Based on such a special design, the high thermal conductivity helps the cooler friction layer dissipate the heat getting from the hotter part during the contact process through the cooling system, and T_c decreases with the thermal conductivity of the friction layer. When the temperature of the heating system and the cooling system maintains at 593.15 K and 283.15 K, respectively, T_c is 396 K when the thermal conductivity of the cooler friction layer is 0.01 W m^{-1} K^{-1}, and T_c decreases to 277 K when the thermal conductivity of the cooler friction layer is 1 W m^{-1} K^{-1}.

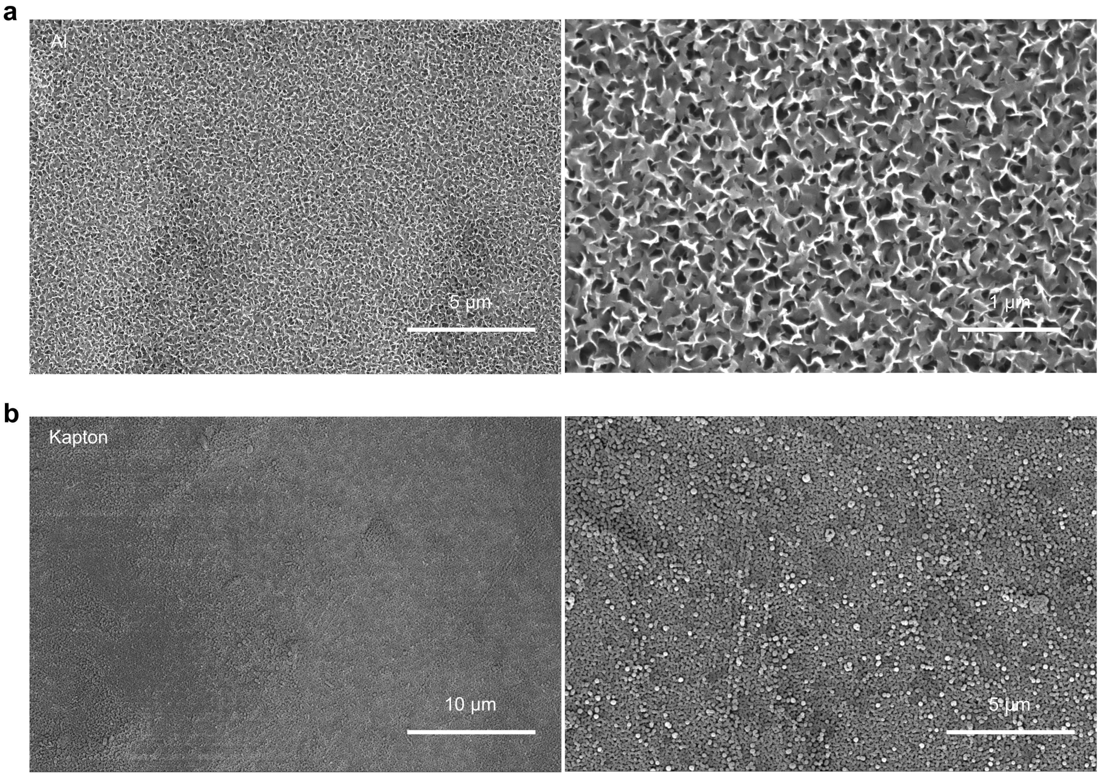

Supplementary Fig. 3 Scanning electron microscope (SEM) images of the chemical reactive etched Al foil and the reactive ion etched Kapton film. a The SEM images of the chemical reactive etched Al foil. Scale bars: 5 μm (left) and 1 μm (right). **b** The SEM images of the reactive ion etched Kapton film. Scale bars: 10 μm (left) and 5 μm (right).

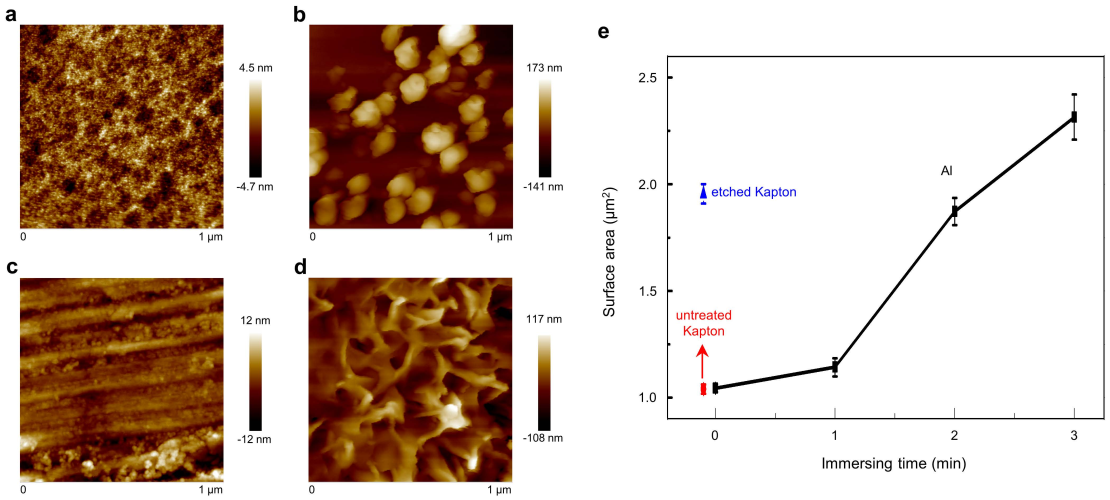

Supplementary Fig. 4 Characterization of the surface morphology of the friction layers. a Atomic force microscopy (AFM) topography of an untreated Kapton. **b** AFM topography of a reactive ion etched Kapton. **c** AFM topography of an untreated Al foil. **d** AFM topography of a chemical reactive etched Al foil. **e** Surface area of different friction layers. Scanning area, 1 μm^2. Error bars represent the mean ± standard deviation of five independent experiments.

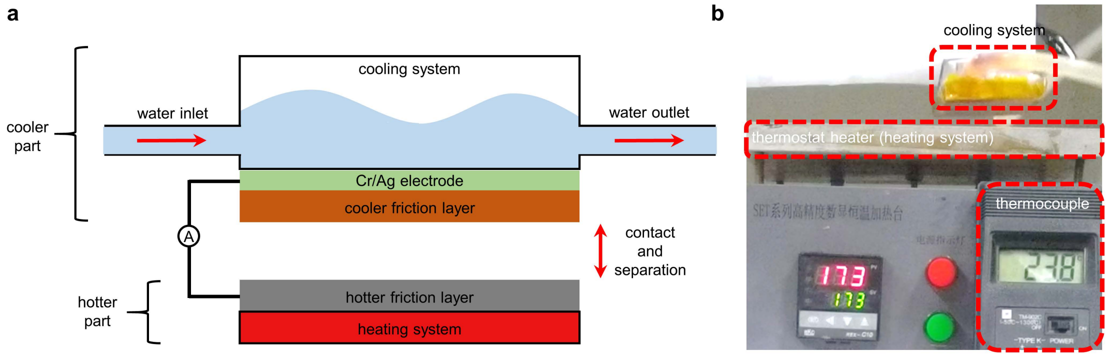

Supplementary Fig. 5 The schematic diagram and optical photograph of TDNG in experiments. a The schematic diagram of TDNG in experiments. **b** The optical photograph of TDNG in experiments.

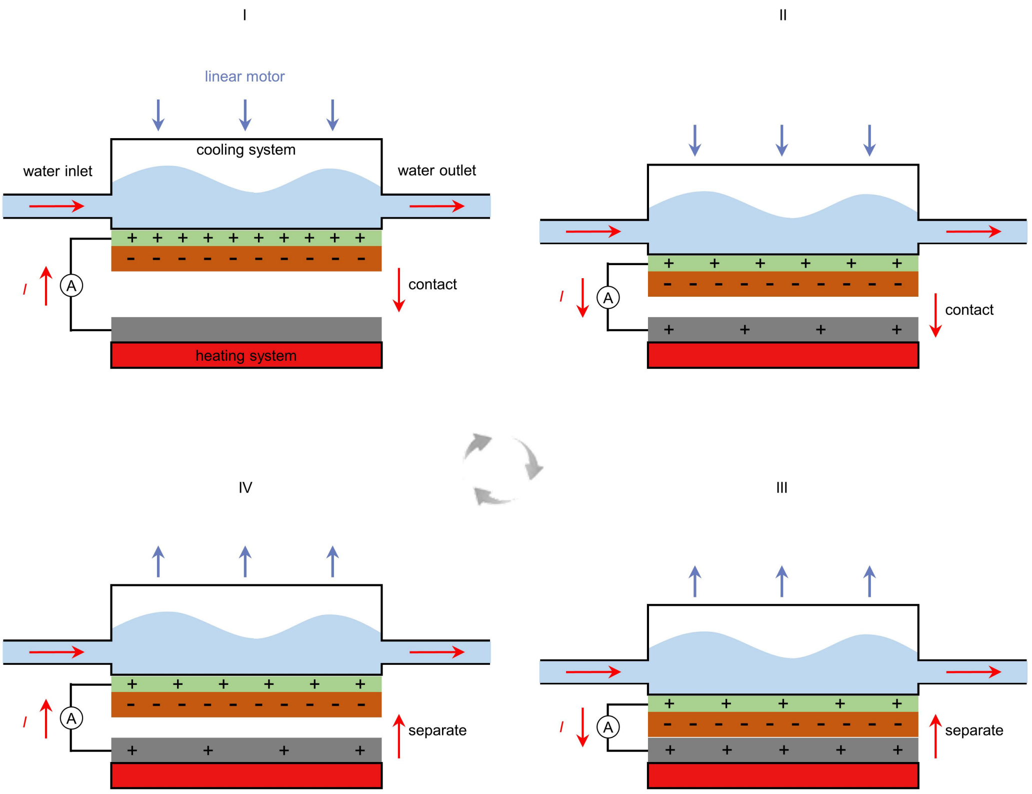

Supplementary Fig. 6 The working mechanism of TDNG. With a periodic change of working distance from I to IV, and IV to I, the amount of induced charges in Cr/Ag electrodes changes periodically, which produces an alternating current between electrodes.

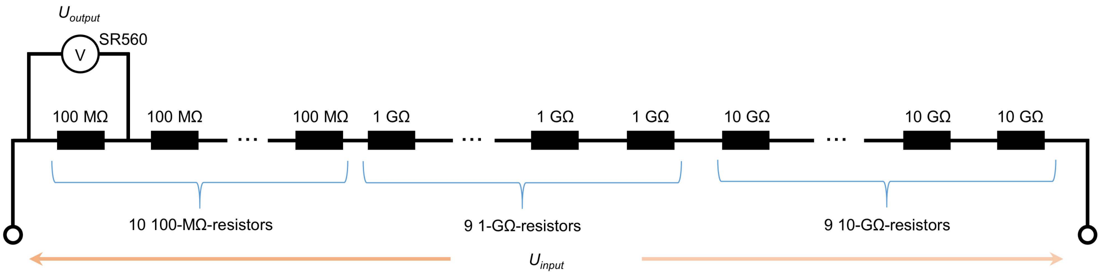

Supplementary Fig. 7 The schematic diagram of the voltage measurement using SR560 and voltage divider. The voltage of TDNG is measured by SR560 and a voltage divider. The largest measuring range of SR560 is ±3 V. To measure the output voltage of TDNG up to thousands of volts, a voltage divider (voltage divider is a passive linear circuit that produces an output voltage that is a fraction of its input voltage) is used. The voltage divider is composed of ten 100-MΩ-resistors, nine 10-GΩ-resistors, and nine 1-GΩ-resistors, whose whole resistance is 100 GΩ. The total resistance of 100 GΩ is designed to match the open-circuit resistance of TDNG. When the external resistance is greater than 30 GΩ, the end voltage is consistent with the open-circuit voltage. The 100-MΩ-resistor (R_1) in the voltage divider is used to match the input impedance of SR560 (100 MΩ). When the output voltage of TDNG (U_{input}) is applied across the series resistance and the output is the voltage across R_1. Since the current (I) flowing through each resistor in the series circuit is equal, the relationship between the input voltage (U_{input}) and the output voltage (U_{output}) is:

$$U_{output} = \frac{R_1}{R_{\mathrm{w}}} U_{input} \tag{S9}$$

where R_{w} is the whole resistance of series resistances. Because the whole resistance of series resistances is 100 GΩ, R_1 is 100 MΩ, and the output voltage of TDNG (U_{input}) can be obtained as follows

$$U_{input} = 1000 \times U_{output} \tag{S10}$$

In this way, the measurement range of SR560 can be enhanced to ±3000 V, and the output voltage of TDNG up to thousands of volts can be measured.

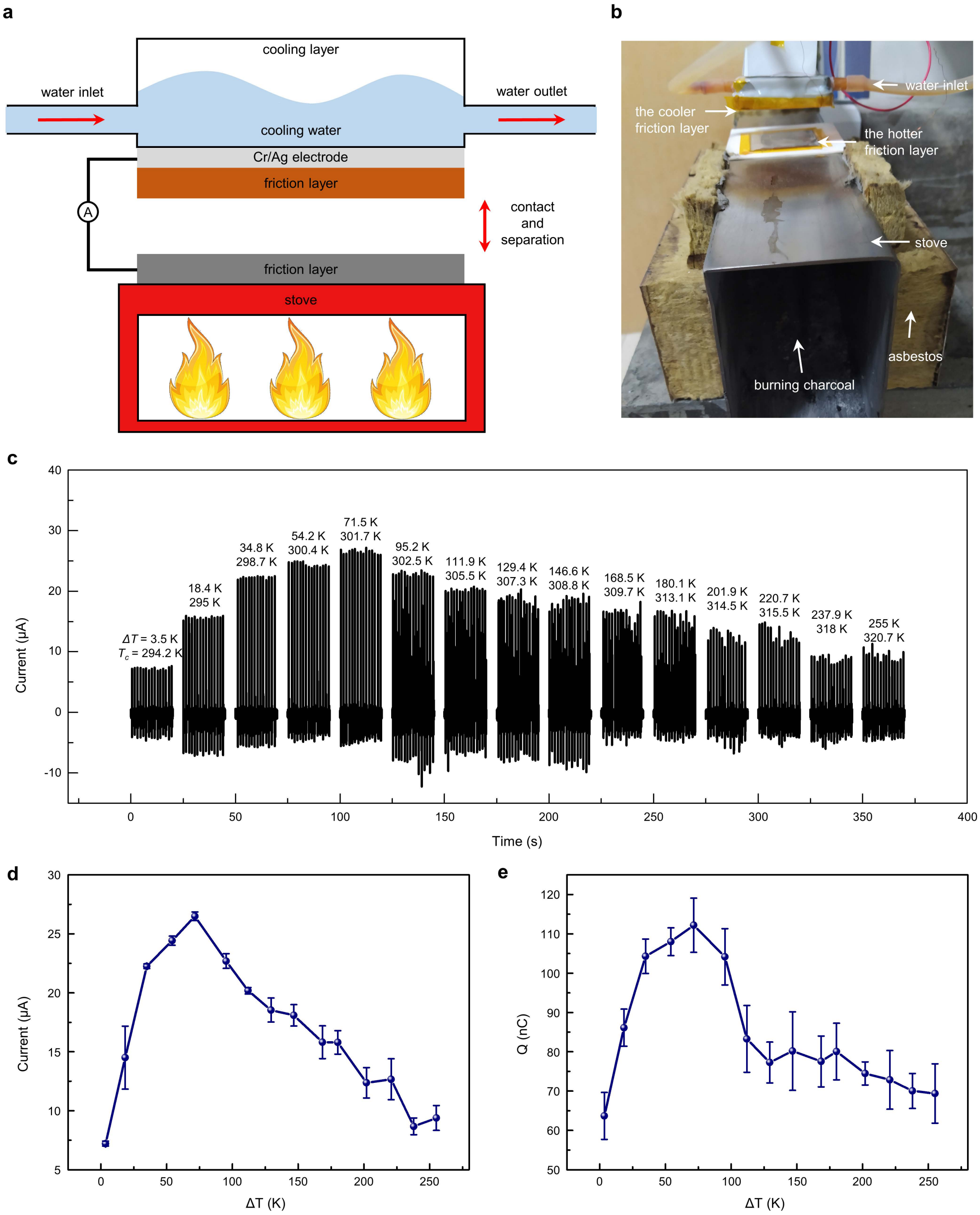

Supplementary Fig. 8 The relationship between the output of TDNG and Δ*T* without using the electric heating/cooling system. a The schematic diagram of the TDNG using the stove as a heating system. **b** The optical photograph of the real TDNG using the stove as a heating system. **c** The output current curve of TDNG with the increase of Δ*T*. **d** The peak current of TDNG with the increase of Δ*T*. Error bars represent the mean ± standard deviation of three independent experiments. **e** The transferred charge per cycle *Q* of TDNG with the increase of Δ*T*. Error bars represent the mean ± standard deviation of three independent experiments. As shown in

Supplementary Fig. 8a-b, we made a wood-burning/charcoal stove as a heating system consisting of a steel rectangular tube and asbestos. An insulating corundum plate is added between the friction layer and stove to avoid the possible electrostatic induction from the stove. When the burning charcoal is added to the stove, the highest temperature of the Al friction layer can reach ~600 K. The cooler friction layer of the TDNG is cooled by a water cooling system without electrostatic induction. In this way, the output of TDNG with different ΔT are obtained (Supplementary Fig. 8c). As Supplementary Fig. 8d demonstrated, when the temperature of the hotter friction layer is 373.2 K (±2 K), the temperature of the cooler friction layer is 301.7 K, and the temperature difference is 71.5 K (±2 K), the largest short-circuit current of TDNG can reach 26.49±0.36 μA, which is 3.66 times of that temperature difference equals 3.5 K (7.21±0.22 μA). Besides, at this situation, as shown in Supplementary Fig. 8e, the transferred charge per cycle is the maximum 112.18±6.88 nC, which is 1.76 times of the transferred charge per cycle 63.68±5.97 μC at temperature difference 3.5 K and cooler friction layer's temperature 294.2 K.

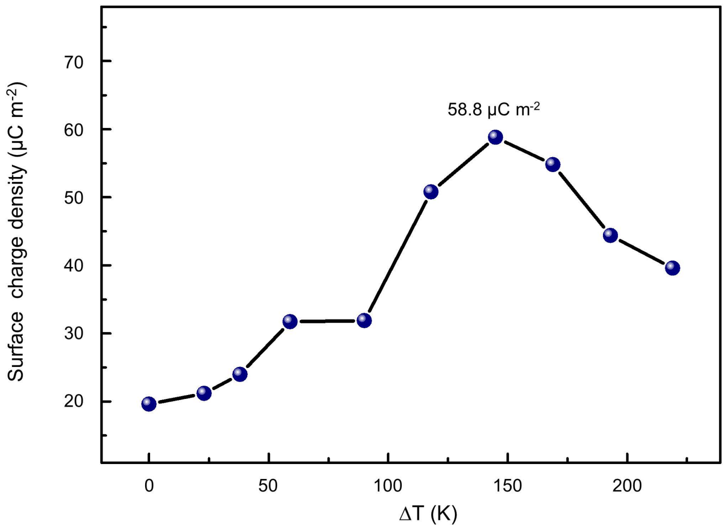

Supplementary Fig. 9 The surface charge density at different Δ*T*. As Δ*T* increases, the surface charge density of TDNG increases firstly and then decreases. When Δ*T* equals 145 K, the largest surface charge density can reach 58.8 μC m^{-2}, which is 3 times that of Δ*T* equals to 0 K (19.6 μC m^{-2}).

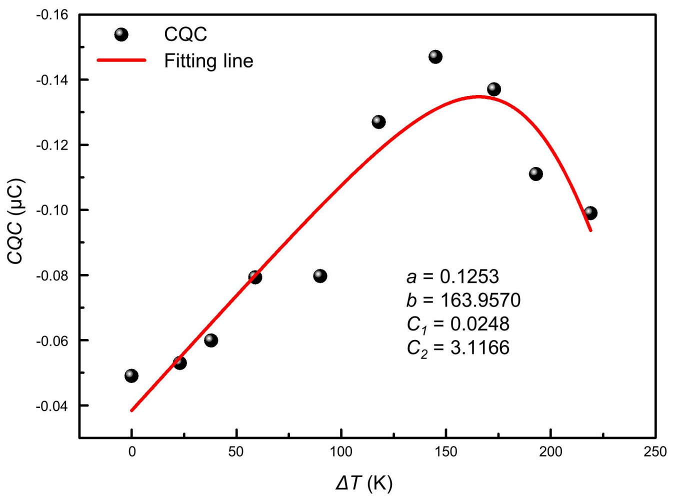

Supplementary Fig. 10 The fitting analysis of the transferred charge quantities per cycle (*CQC*) at short-circuit condition. According to the theoretical study and assuming that the transferred charge equals to accumulated charge in the friction layer (Supplementary Note 1), the relationship between CQC of TDNG and temperature difference *ΔT* follows

$$CQC = (-C_1\frac{\Delta T+b}{1-a} + C_2)e^{-SAt_0}A\left[1-\left(1+\frac{d}{RA\varepsilon_0\varepsilon_r}\int_0^t e^{\frac{1}{RA\varepsilon_0}\left(\frac{d}{\varepsilon_r}t+\int_0^t x_{(t)}dt\right)}dt\right)e^{-\frac{1}{RA\varepsilon_0}\left(\frac{d}{\varepsilon_r}t+\int_0^t x_{(t)}dt\right)}\right] \quad \text{(S11)}$$

A nonlinear fit to CQC under different *ΔT* has been performed (red line). The obtained material-related correction factor *a* equals to 0.1253, and the material-related correction factor *b* equals to 163.957. The obtained material-related correction factor C_1 equals to 0.0248, and C_2 equals to 3.1166.

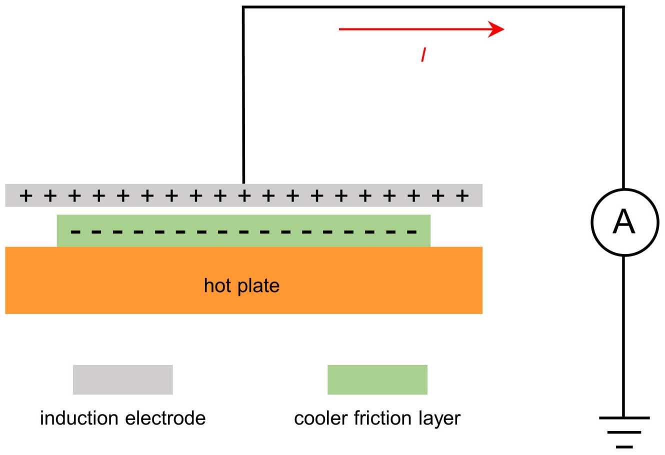

Supplementary Fig. 11 Schematic diagram of the open-circuit thermally stimulated discharge of the cooler friction layer. When the temperature of the cooler friction layer increases, the accumulated charge in the cooler friction layer will release, and the induced charge in the induction electrode will decrease through flowing into the ground, where the change of TSD current can be tested by an ammeter.

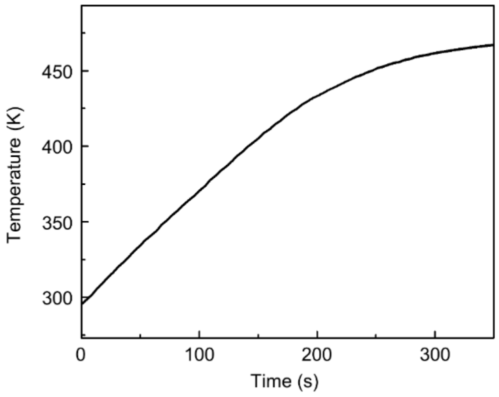

Supplementary Fig. 12 Temperature rising curves of the heating system in TSD testing. Temperature of the heating system is elevated from 295 K to 467 K in 360 s.

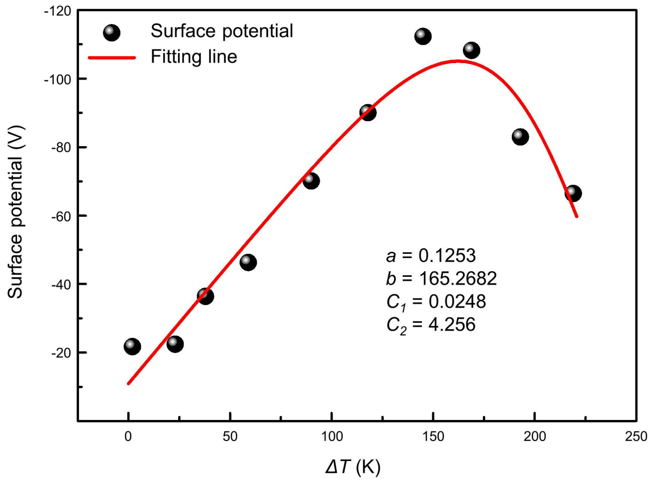

Supplementary Fig. 13 The fitting analysis of the Surface potential V_s. Based on the relationship between the surface charge density and surface potential:

$$\sigma_s = \frac{\varepsilon_0\varepsilon_r V_s}{d_0} \tag{S12}$$

Combining equation (S4), the surface potential V_s can be derived as follows

$$V_s = \frac{d_0\left(-C_1\frac{\Delta T+b}{1-a}+C_2\right)e^{-SAt_0}}{\varepsilon_r\varepsilon_0}\left[1-\left(1+\frac{d}{RA\varepsilon_0\varepsilon_r}\int_0^t e^{\frac{1}{RA\varepsilon_0}\left(\frac{d}{\varepsilon_r}t+\int_0^t x_{(t)}dt\right)}dt\right)e^{-\frac{1}{RA\varepsilon_0}\left(\frac{d}{\varepsilon_r}t+\int_0^t x_{(t)}dt\right)}\right] \tag{S13}$$

where d_0 is the distance between the electrostatic voltmeter and the friction layer. According to equation (S13), a nonlinear fit between the surface potential of the cooler friction layer and temperature differences has been performed. The obtained material-related correction factor a equals to 0.1253, b equals to 165.2682, C_1 equals to 0.0248, and C_2 equals to 4.256.

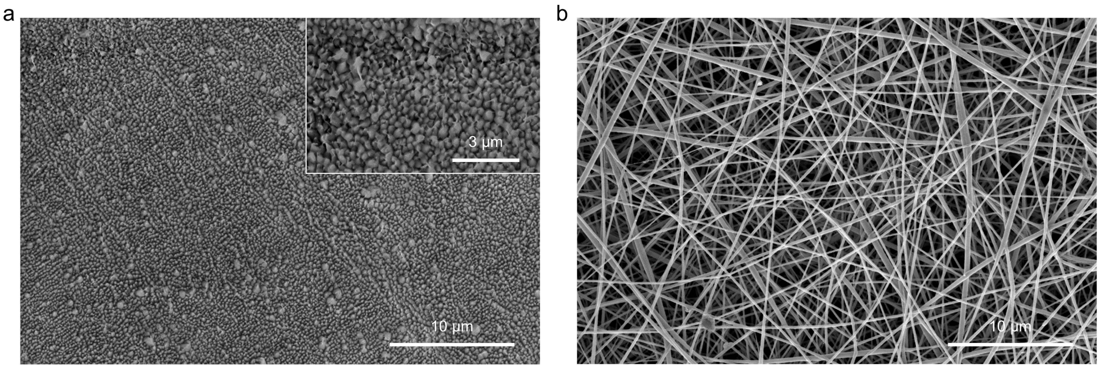

Supplementary Fig. 14 Scanning electron microscope images of the nanostructure of **a** PTFE and **b** PA-6. Scale bars: 10 μm (**a**), 3 μm (inset of **a**), and 10 μm (**b**).

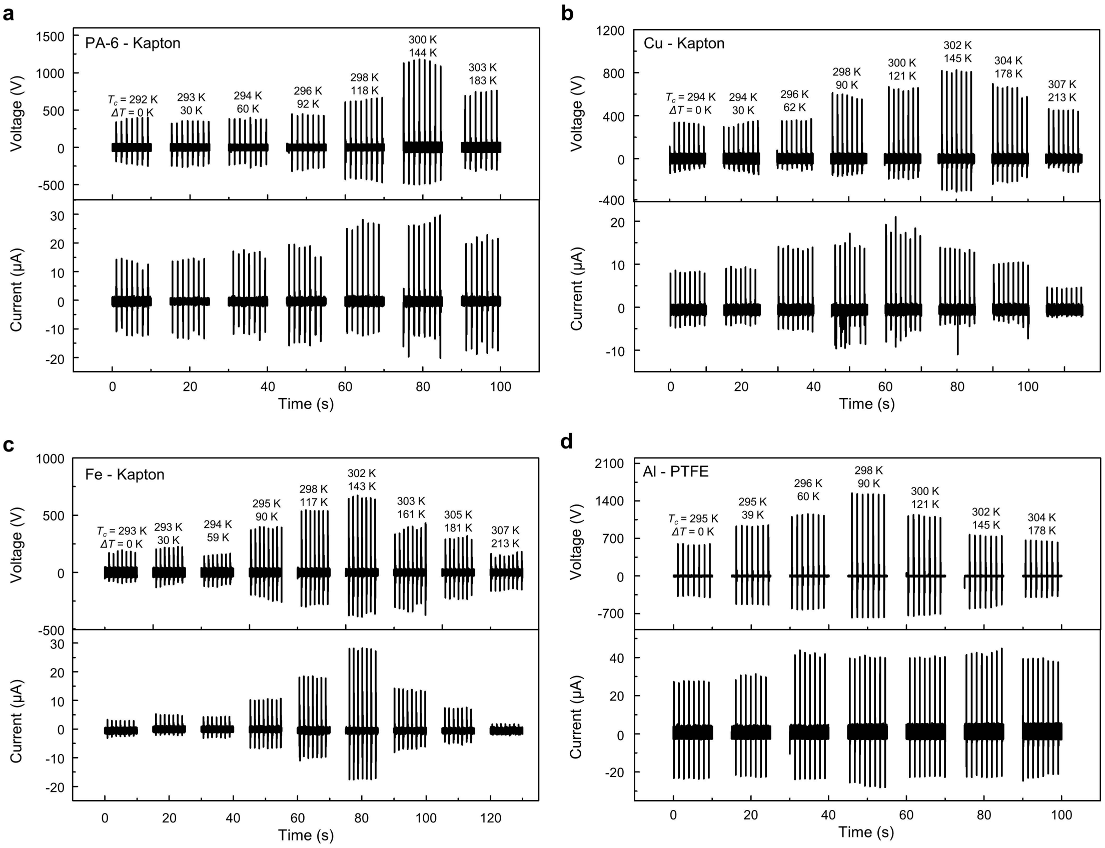

Supplementary Fig. 15 The open-circuit voltage and the short-circuit current of different TDNGs under different temperature difference Δ*T*. **a** The open-circuit voltage and the short-circuit current of the PA-6-Kapton TDNG under different Δ*T*. T_c in figure is the temperature of the cooler friction layer. **b** The open-circuit voltage and the short-circuit current of the Cu-Kapton TDNG under different Δ*T*. **c** The open-circuit voltage and the short-circuit current of the Fe-Kapton TDNG under different Δ*T*. **d** The open-circuit voltage and the short-circuit current of the Al-PTFE TDNG under different Δ*T*.

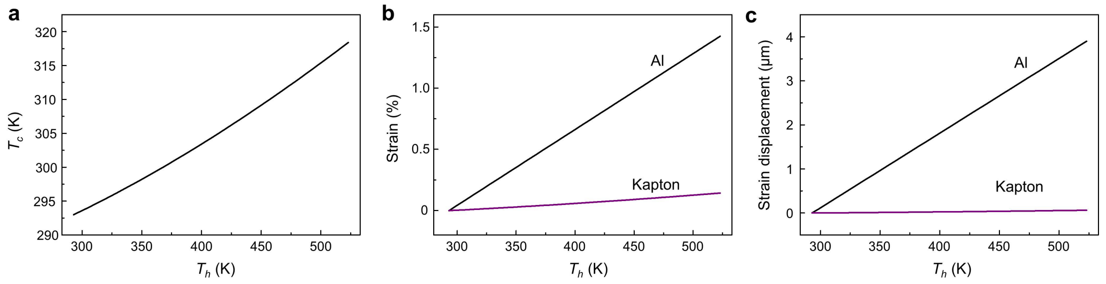

Supplementary Fig. 16 The strain of TDNG when the temperature of the hotter friction layer T_h increases from 293 K to 523 K. a The relationship between the temperature of the cooler friction layer T_c and T_h. Theoretically, the change of T_c is just 24 K with T_h increases from 293 K to 523 K. **b** The strain of Al and Kapton when T_h increases from 293 K to 523 K. The volume strain of Kapton is just 0.14% (purple line), and the volume strain of Al is 1.4% (black line). **c** The displacement of Al and Kapton when T_h increases from 293 K to 523 K. The displacement of Kapton is 0.1 μm (purple line), and the displacement of Al is 3.9 μm (black line). Compared to the whole friction layer, the deformation is very small and can be ignored.

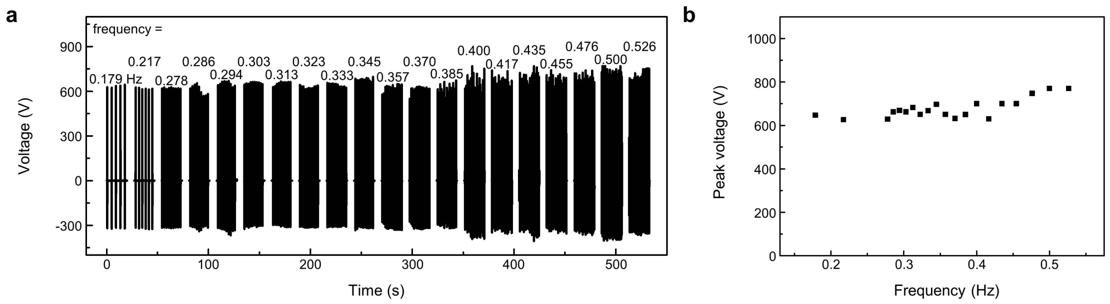

Supplementary Fig. 17 The relationship between the driving frequency and the output voltage of TDNG. a The voltage of TDNG under different frequencies (from 0.179 Hz to 0.526 Hz). **b** The peak voltage under different frequencies. When the working frequency increases from 0.179 Hz to 0.526 Hz, the largest open-circuit voltage of TDNG is increased from 647 V to 770 V.

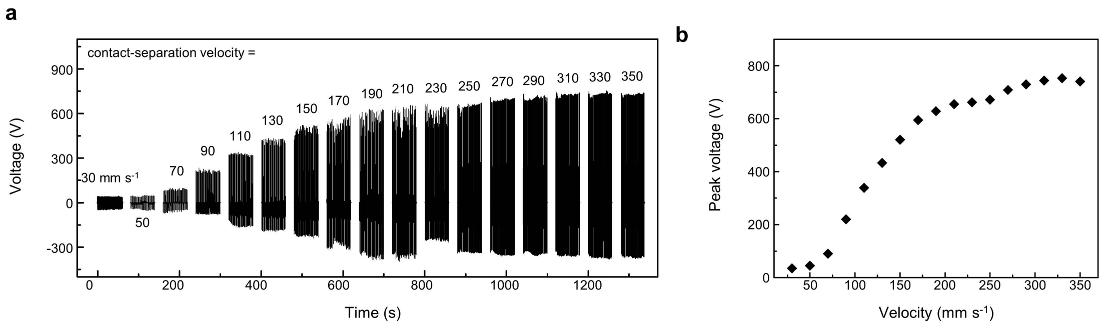

Supplementary Fig. 18 The relationship between contact-separated velocity and the output voltage of TDNG. a The voltage of TDNG under different contact-separation velocity (from 30 mm s^{-1} to 350 mm s^{-1}). **b** The peak voltage under different contact-separation velocity. When the contact-separation velocity (*v*) increases from 30 mm s^{-1} to 350 mm s^{-1}, the open-circuit voltage of TDNG is enhanced from 35 V to 753 V.

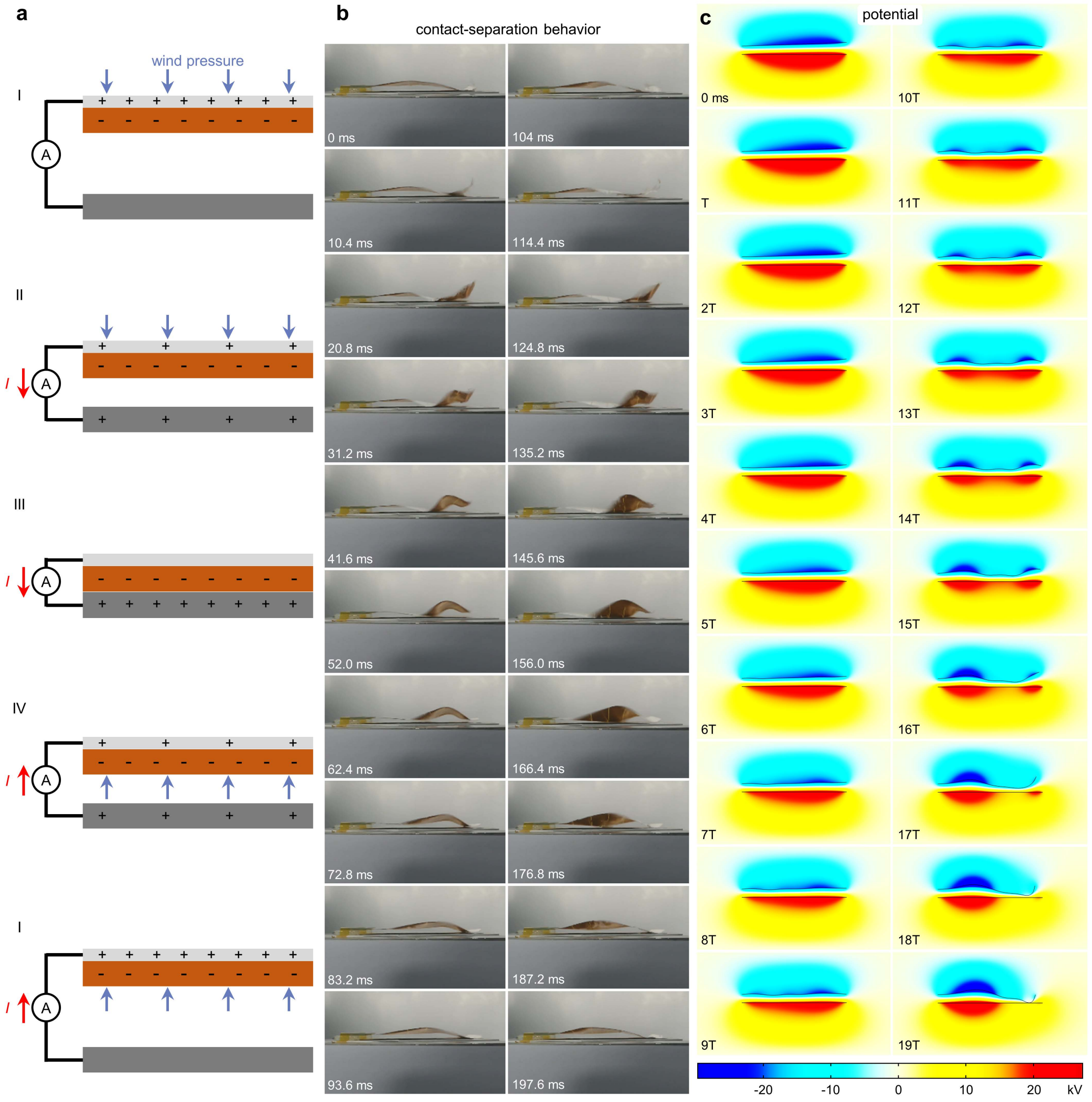

Supplementary Fig. 19 The schematic diagram and pictures of an operational wind-driven TDNG. a The schematic diagram of the working mechanism of the wind-driven TDNG. Under the action of the wind pressure, two friction layers contact and separate periodically. With a periodic contact-separation process from I to IV, and IV to I, the number of induced charges in electrodes changes periodically, which produces an alternating current between electrodes. **b** Sequence optical photographs of an operational wind-driven TDNG taken by a high-speed camera. Under the wind, Kapton film shows a motion like mechanical wave transmission and illustrate different states. Near the fixed end, the amplitude is nearly zero with only a very small-amplitude oscillation arising. In contrast, near the free end, a traveling wave propagates toward the free end with increasing amplitude. When the Kapton film contacts Al foil, triboelectrification occurs, and

the wind energy is converted to electricity. **c** The potential distribution of an operational wind-driven TDNG in difference times (T = 7.5 ms).

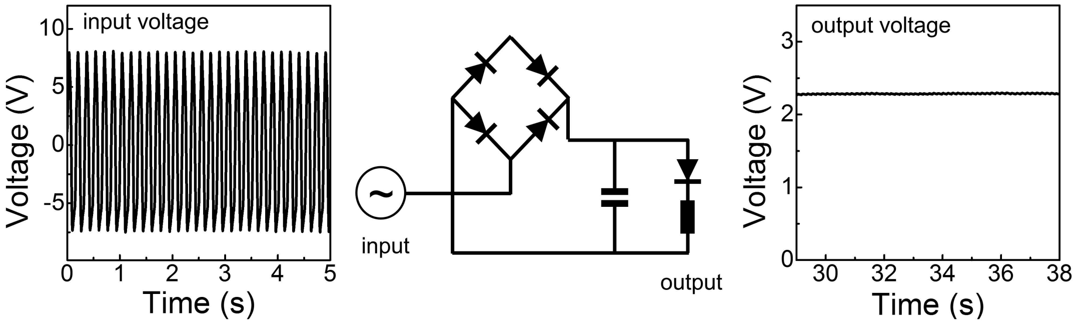

Supplementary Fig. 20 Schematic diagram of the filter circuit. The alternating current input can be converted to a constant voltage/current output through a filter circuit.

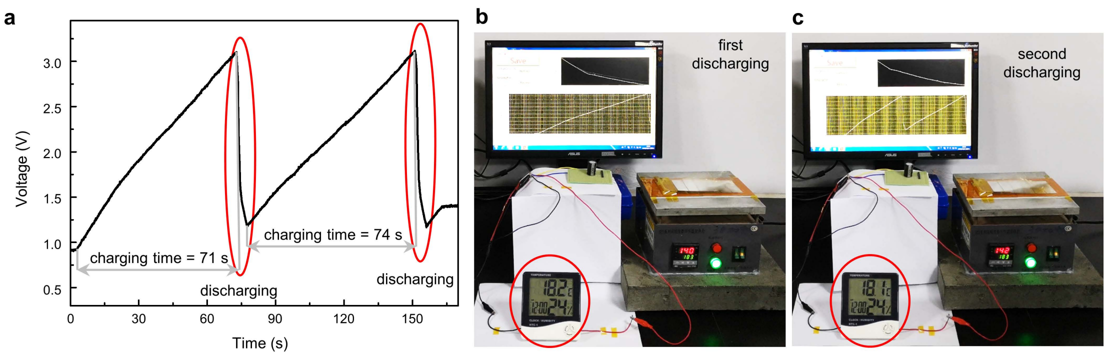

Supplementary Fig. 21 Powering for the temperature-humidity sensor. a The charging curve of the temperature-humidity sensor. The storage time is marked in two charging-discharging cycles, where the first charging time is 71 s, and the second charging time is 74 s. The average charging time of 73 s is calculated with five charging-discharging cycles. **b** The optical photograph of the first discharging process. **c** The optical photograph of the second discharging process.

Supplementary Table 1 The electrical performance of previous wind-driven TENGs and that of our work.

**voltage (V)**	**current (μA)**	**wind speed ($m\ s^{-1}$)**	**References**
60	2	10	[3]
98	16.3	27	[4]
50	45	22	[5]
396	75	18.4	[6]
250	70	22	[7]
36	11.8	17.9	[8]
135	12	24.6	[9]
140	1	15	[10]
63.3	15	20	[11]
50	15	10	[12]
21<br>1.25	-<br>0.14	25<br>3	[13]
150<br>-	70<br>150	7<br>10	[14]
-	12.5	15.8	[15]
1000	90	14	[16]
320	27	17	[17]
-	17.5	17.3	[18]
200	20	20	[19]
39<br>49	3<br>5	10<br>6.8	[20]
375<br>-<br>-	248<br>326<br>73	14.5<br>20<br>3	[21]
110	80	10	[22]
10.7<br>-	-<br>-	2.7<br>4.9	[23]
-	24	18	[24]
12	1.8	22.5	[25]
427	123.3	5	This work

Supplementary References

1. Niu, S. et al. Theoretical study of contact-mode triboelectric nanogenerators as an effective power source. *Energy Environ. Sci.* **6**, 3576-3584 (2013).
2. Jiang, T. et al. Theoretical study on rotary-sliding disk triboelectric nanogenerators in contact and non-contact modes. *Nano Res.* **9**, 800-807 (2016).
3. Xu, M. et al. An aeroelastic flutter based triboelectric nanogenerator as a self-powered active wind speed sensor in harsh environment. *Extreme Mech. Lett.* **15**, 122-129 (2017).
4. Zhang, L. et al. Lawn structured triboelectric nanogenerators for scavenging sweeping wind energy on rooftops. *Adv. Mater*. **28**, 1650-1656 (2016).
5. Zhao, Z. et al. Freestanding flag-type triboelectric nanogenerator for harvesting high-altitude wind energy from arbitrary directions. *ACS Nano* **10**, 1780-1787 (2016).
6. Quan, Z., Han, C.B., Jiang, T. & Wang, Z. L. Robust thin films-based triboelectric nanogenerator arrays for harvesting bidirectional wind energy. *Adv. Energy Mater.* **6**, 1501799 (2016).
7. Bae, J. et al. Flutter-driven triboelectrification for harvesting wind energy. *Nat. Commun*. **5**, 4929 (2014).
8. Su, Y. et al. Segmented wind energy harvester based on contact-electrification and as a self-powered flow rate sensor. *Chem. Phys. Lett.* **653**, 96-100 (2016).
9. Gao, T., Zhao, K., Liu, X. & Yang, Y. Implanting a solid Li-ion battery into a triboelectric nanogenerator for simultaneously scavenging and storing wind energy. *Nano Energy* **41**, 210-216 (2017).
10. Wang, J. et al. Smart network node based on hybrid nanogenerator for self-powered multifunctional sensing. *Nano Energy* **33**, 418-426 (2017).
11. Dudem, B. et al. Nanopillar-array architecture PDMS-based triboelectric nanogenerator integrated with a windmill model for effective wind energy harvesting. *Nano Energy* **42**, 269-281 (2017).
12. Jeon, S. B. et al. Self-powered electro-coagulation system driven by a wind energy harvesting triboelectric nanogenerator for decentralized water treatment. *Nano Energy* **28**, 288-295 (2016).

13. Seol, M. L. et al. Vertically stacked thin triboelectric nanogenerator for wind energy harvesting. *Nano Energy* **14**, 201-208 (2015).
14. Feng, Y. et al. Leaves based triboelectric nanogenerator (TENG) and TENG tree for wind energy harvesting. *Nano Energy* **55**, 260-268 (2019).
15. Park, S. J. et al. A multi-directional wind based triboelectric generator with investigation of frequency effects. *Extreme Mech. Lett.* **19**, 46-53 (2018).
16. Liu, X., Zhao, K. & Yang, Y. Effective polarization of ferroelectric materials by using a triboelectric nanogenerator to scavenge wind energy. *Nano Energy* **53**, 622-629 (2018).
17. Bian, Y. et al. Triboelectric nanogenerator tree for harvesting wind energy and illuminating in subway tunnel. *Adv. Mater. Technol.* **3**, 1700317 (2018).
18. Guo, H. et al. A nanogenerator for harvesting airflow energy and light energy. *J. Mater. Chem. A* **2**, 2079-2087 (2014).
19. Jiang, Q., Chen, B., Zhang, K. & Yang, Y. Ag nanoparticle-based triboelectric nanogenerator to scavenge wind energy for a self-charging power unit. *ACS Appl. Mater. Interfaces* **9**, 43716-43723 (2017).
20. Dudem, B., Kim, D. H. & Yu, J. S. Triboelectric nanogenerators with gold-thin-film-coated conductive textile as floating electrode for scavenging wind energy. *Nano Res.* **11**, 101-113 (2017).
21. Cui, S., Zheng, Y., Liang, J. & Wang, D. Triboelectrification based on double-layered polyaniline nanofibers for self-powered cathodic protection driven by wind. *Nano Res.* **11**, 1873-1882 (2018).
22. Yang, Y. et al. Triboelectric nanogenerator for harvesting wind energy and as self-powered wind vector sensor system. *ACS Nano* **7**, 9461–9468 (2013).
23. Zheng, L. et al. A hybridized power panel to simultaneously generate electricity from sunlight, raindrops, and wind around the clock. *Adv. Energy Mater.* **5**, 1501152 (2015).
24. Su, Y. et al. Wind energy harvesting and self-powered flow rate sensor enabled by contact electrification. *J. Phys. D: Appl. Phys.* **49**, 215601 (2016).
25. Yong, H. et al. Highly reliable wind-rolling triboelectric nanogenerator operating in a wide wind speed range. *Sci. Rep.* **6**, 33977 (2016).
