# Supplementary material for: High performance temperature difference triboelectric nanogenerator
Source: Nat Commun. 2021 Aug 6;12:4782. doi: 10.1038/s41467-021-25043-2 (PMC8346487; doi:10.1038/s41467-021-25043-2)
Supplement: Supplementary file 3 — Description of Additional Supplementary Files [file 41467_2021_25043_MOESM3_ESM.pdf]

Description of Additional Supplementary Files

Supplementary Movie 1

Legend: The driving and testing of the temperature difference triboelectric nanogenerator with controllable friction layer temperature (TDNG).

Supplementary Movie 2

Legend: Harvesting the mechanical energy of human tapping using TDNG.

Supplementary Movie 3

Legend: The working behavior of the wind-driven TDNG.

Supplementary Movie 4

Legend: 955 white LEDs lighted by the wind-driven TDNG under a wind speed of ~8.3 m s^{-1}.

Supplementary Movie 5

Legend: The red LED, blue LED, and white LED are lighted sequentially and continuously by the wind-driven TDNG when the temperature difference changed from 0 K to 40 K.

Supplementary Movie 6

Legend: A temperature-humidity sensor is powered by the wind-driven TDNG.
